# Biomarker Discovery for Immunotherapy of Pituitary Adenomas: Enhanced Robustness and Prediction Ability by Modern Computational Tools

**DOI:** 10.3390/ijms20010151

**Published:** 2019-01-03

**Authors:** Qingxia Yang, Yunxia Wang, Song Zhang, Jing Tang, Fengcheng Li, Jiayi Yin, Yi Li, Jianbo Fu, Bo Li, Yongchao Luo, Weiwei Xue, Feng Zhu

**Affiliations:** 1 School of Pharmaceutical Sciences, Chongqing University, Chongqing 401331, China; yangqx@cqu.edu.cn (Q.Y.); 20132902008@cqu.edu.cn (J.T.); libcell@cqu.edu.cn (B.L.); 2 College of Pharmaceutical Sciences, Zhejiang University, Hangzhou 310058, China; lfwyx@zju.edu.cn (Y.W.); zhangsong_@zju.edu.cn (S.Z.); lifengcheng@zju.edu.cn (F.L.); yinjiayi@zju.edu.cn (J.Y.); liyi@email.com (Y.L.); fujianbo@zju.edu.cn (J.F.); luo.yongchao@foxmail.com (Y.L.)

**Keywords:** pituitary adenomas, immunotherapy, immune-related gene markers, transcriptomics

## Abstract

Pituitary adenoma (PA) is prevalent in the general population. Due to its severe complications and aggressive infiltration into the surrounding brain structure, the effective management of PA is required. Till now, no drug has been approved for treating non-functional PA, and the removal of cancerous cells from the pituitary is still under experimental investigation. Due to its superior specificity and safety profile, immunotherapy stands as one of the most promising strategies for dealing with PA refractory to the standard treatment, and various studies have been carried out to discover immune-related gene markers as target candidates. However, the lists of gene markers identified among different studies are reported to be highly inconsistent because of the greatly limited number of samples analyzed in each study. It is thus essential to substantially enlarge the sample size and comprehensively assess the robustness of the identified immune-related gene markers. Herein, a novel strategy of direct data integration (DDI) was proposed to combine available PA microarray datasets, which significantly enlarged the sample size. First, the robustness of the gene markers identified by DDI strategy was found to be substantially enhanced compared with that of previous studies. Then, the DDI of all reported PA-related microarray datasets were conducted to achieve a comprehensive identification of PA gene markers, and 66 immune-related genes were discovered as target candidates for PA immunotherapy. Finally, based on the analysis of human protein–protein interaction network, some promising target candidates (*GAL*, *LMO4*, *STAT3*, *PD-L1*, *TGFB* and *TGFBR3*) were proposed for PA immunotherapy. The strategy proposed together with the immune-related markers identified in this study provided a useful guidance for the development of novel immunotherapy for PA.

## 1. Introduction

Pituitary adenoma (PA) accounts for approximately 8–15% of intracranial tumors [1,2,3] and its prevalence is estimated to be ~14–22% by autopsy and radiological studies [4,5,6]. Due to its aggressive infiltration into the surrounding brain structure and its severe complications (such as Cushing’s disease, hyperprolactinemia, and acromegaly), the effective managements of PA are still required [7,8,9], which involves trans-sphenoidal surgery, radiotherapy, and medicine [10]. Trans-sphenoidal surgery is reported as sometimes contraindicated or ineffective [11], and the application of radiotherapies is greatly limited by its subsequent hypopituitarism [11]. So far, medical therapy has been expected and emerged to provide the treatment of enhanced efficacy, safety and tolerance [11,12,13], and several drugs have already been approved by U.S. FDA (Food and Drug Administration) for treating certain complications of PA [14,15,16]. For example, cabergoline, octreotide, and pasireotide have FDA approval for the management of hyperprolactinemia, acromegaly, and Cushing’s disease, respectively [17,18,19,20]. Apart from the drugs aiming at PA complication, temozolomide is found to penetrate well into central nervous system, and therefore has been tested in clinical trial (Phase II) for treating PAs [21,22,23,24].

However, those approved therapeutics primarily focus on managing the complication of PA rather than PA itself [17,18], which are incapable of removing the tumor cells and are ineffective in turning the pituitary back to its normal state [25]. The therapeutics designed for the direct treatment of PA are primarily chemotherapy [21], kinase inhibitor [26,27,28,29], and monoclonal antibody [30,31,32]. Till now, chemotherapy and kinase inhibitors have emerged as the most promising candidates with temozolomide and lapatinib in clinical developments [21,26,33], but they suffer from severe myelotoxicity, mental disturbances or cardiac dysrhythmia that can substantially affect the approval processes of these clinical candidates [34,35,36]. So far, no effective drug has been developed for treating non-functional pituitary tumors, and the removal of cancerous cells directly from the pituitary is still under experimental investigation [37,38,39]. It is thus crucial to discover a new strategy for PA treatment. With the advent of immunotherapy [40,41], the management of PA base for this new strategy has become the focus of current research [1,30]. In other words, due to its high specificity and good safety profiles [42], immunotherapy stands as one of the most promising alternatives for treating PAs that are resistant or refractory to the standard treatments [43,44,45,46].

Although no immunotherapy of PA has been approved yet, various studies have been carried out to discover immune-related genes or target candidates. Particularly, as an immunomodulatory peptide, protein galanin (*GAL*) is substantially suppressed in the tissue of PA patients compared with those in healthy pituitaries [47]. Increased levels (both mRNA and protein) of programmed death-ligand 1 (*PD-L1*, the immunosuppressive protein) are observed in tumor tissue of PA patients [43], which can induce immune evasion by desensitizing the recognition and elimination of cancer cells via CD8^+^ lymphocytes [48]. Transforming growth factor-beta (*TGFB*) was discovered as one of the chief immune suppressive mediators of prolactin (PRL) secretions and PA proliferation [49,50]. Apart from the above studies, a variety of transcriptomics analyses (demonstrated in Table 1) have been conducted to identify the differentially expressed genes (DEGs) between PA patients and normal ones [51,52,53,54]. Due to their Omics-based nature, these analyses can accelerate more effectively the discovery of target candidates compared with previous studies. However, because of the limited number of both the patients and controls in each analysis (~4–16 samples for patients and ~0–9 for healthy individuals shown in Table 1), the lists of DEGs identified from different analyses are reported to be highly inconsistent [55,56], which asks for substantial enhancements of the robustness of the identified PA biomarkers [57,58]. Moreover, no Omics study has been conducted to reveal immune-related genes and mechanisms underlying PA’s development, which is essential for the discovery of promising targets for PA immunotherapy [59]. Thus, it is urgently needed to systematically identify the immune-related DEGs based on the comprehensive analysis of the PA-based Omics datasets with significantly enlarged sample size (for both PA patients and healthy individuals) [60].

In this study, a strategy of direct data integration (DDI) was proposed to combine available PA microarray datasets for significantly enlarging the sample size. To test the impact of the DDI strategy on the classification ability and the robustness of identified DEGs, its performance and disease relevance were first evaluated by comparing with previously published datasets. Then, all currently available PA-related microarray datasets were directly integrated to achieve comprehensive identification of DEGs between PA patients and healthy individuals. Finally, the immune-related genes were annotated from DEGs, which could be further studied as target candidates for PA immunotherapy. The strategy proposed together with the immune-related DEGs identified in this study provided a useful guidance for future immunotherapy.

## 2. Results and Discussion

### 2.1. The Level of PA-Relevance of the DEGs Identified by Different Analytical Strategies

**Figure 1:**
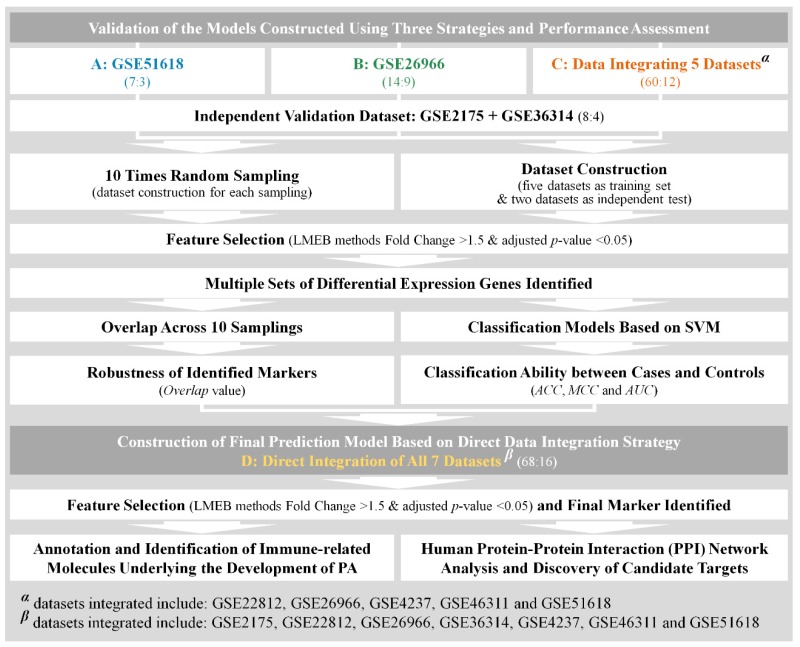
A schematic representation of the direct data integration (DDI) strategy adopted in this study. Four datasets (A: GSE51618, B: GSE26966, C: Data Integrating 5 Datasets and D: Direct Integration of All 7 Datasets) were labeled by blue, green, orange and yellow color, respectively. LMEB: Linear models and empirical Bayes; ACC: accuracy; MCC: Matthews correlation coefficient; AUC: area under the curve.

Several studies have been conducted to identify the DEG capable of distinguishing PA patients from healthy people [51,52,53]. Due to their limited number of disease and healthy samples, the DEGs identified in different studies are reported to be highly inconsistent [55], which requires substantial enhancement in the robustness of the identified DEGs [57,58]. Thus, it is necessary to evaluate the impact of sample size on the robustness and disease relevance of the identified DEGs. In this study, three analytical strategies were proposed based on the construction of three datasets. As illustrated in Figure 1, the datasets included: (A) GSE51618, (B) GSE26966, (C) DDI of five datasets GSE22812, GSE26966, GSE4237, GSE46311, and GSE51618. Clearly, the sample size of dataset C (60 cases and 12 controls) is significantly larger than that of the remaining two (seven cases and three controls for dataset A; 14 cases and six controls for dataset B). By using this DDI strategy, it is now feasible to discuss the effectiveness of this strategy on enhancing the robustness of identified DEGs and systematically assess the impact of sample size on the resulting DEGs.

As the first assessment, the level of PA relevance was reviewed and discussed for three different analytical strategies. Three lists of DEGs were identified using three different strategies by the linear models and empirical Bayes (LMEB, fold change > 1.5 and adjusted *p*-value < 0.05). Top-ranked DEGs (top five for upregulated and another top five for downregulated) and their relevance to PAs were reviewed by comprehensive literature review. As shown in Supplementary Table S1, 3, 4, and 7 of the top 10 DEGs (identified by datasets A, B, and C, respectively) had been reported to be relevant to PAs. Moreover, 32, 56, and 43 KEGG (Kyoto Encyclopedia of Genes and Genomes) pathways were enriched using DEGs identified from datasets A, B, and C, respectively. The top 10 ranked pathways and their relevance to PA were comprehensively reviewed. As shown in Supplementary Table S2, 3, 4, and 7 of the top 10 ranked pathways (identified by datasets A, B, and C, respectively) had been reported to be closely relevant to PAs. Furthermore, top 10 GO (gene ontology) molecular functions (MFs) and biological processes (BPs) and their relevance to PA were comprehensively reviewed. As shown in Supplementary Tables S3 and S4, 5, 5, and 7 of the top 10 ranked BPs and 4, 4, and 6 of the top 10 ranked MFs (identified by datasets A, B, and C, respectively) had been reported to be relevant to PAs. Overall, these findings indicated that all three strategies used in this study showed a certain level of PA relevance in their identified DEGs. The DDI strategy presented a higher percentage of PA-relevant DEGs, pathways, and GO terms, which indicated the enhanced disease relevance of this strategy comparing to that of the single dataset-based one.

### 2.2. Evaluating the Classification Abilities of the DEGs Identified by Different Analytical Strategies

Classification models are frequently constructed in current transcriptomics studies for predicting samples of various disease status [65,66] or assessing the reliability of identified gene markers [67,68]. The capacity of the constructed classification model was evaluated by various metrics including accuracy (*ACC*), sensitivity (*SEN*), specificity (*SPE*), Matthews correlation coefficient (*MCC*), receiver operating characteristics (*ROC*), and area under ROC curve (*AUC value*) [69,70,71]. As shown in Table 2, there was great variation in each assessment metric among the analytical strategies. Particularly, *ACCs*, *SENs*, *SPEs*, and *MCCs* were in the ranges of ~0.67–0.92, ~0.50–0.88, ~0.75–1.00, and ~0.35–0.84 among strategies, respectively. The metrics *ACC* and *MCC* were frequently used in current Omics study to evaluate correctness [72] and stability [73] of the constructed models. As demonstrated in Table 2, the *ACC* of DDI strategy (0.92) was substantially higher than that of the single dataset-based strategies (both are 0.67). Similar to *ACC*, *MCC* of DDI strategy (0.84) was discovered to be higher than that of the other two (0.35 and 0.50, respectively).

**Figure 2:**
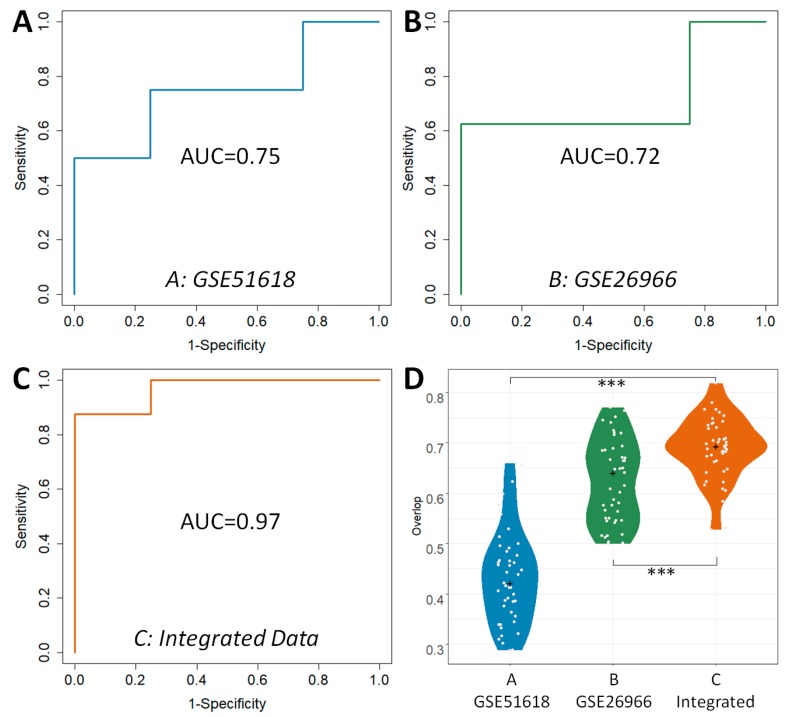
Classification capacity (assessed by *AUC* (area under the curve)) and robustness (evaluated by *Overlap* value) of three classifiers. Classifiers were build using (**A**) GSE51618, (**B**) GSE26966, and (**C**) combination of GSE22812, GSE26966, GSE4237, GSE46311, and GSE51618. Statistical comparative analyses of the *overlap* values among the strategies based on these three datasets were also provided (**D**). The + presents the median of all *overlap* values, and *** presents the *p* value < 0.001.

Apart from *ACC* and *MCC*, the *ROC* and *AUC* were two other popular metrics widely used to assess ability for classification, which were acknowledged to achieve comprehensive performance evaluations. As shown in Figure 2, the *ROC* curves and *AUC* values of three strategies were compared. Grey diagonals represented an invalid model with the corresponding *AUC* value equaled 0.5. As shown in Figure 2, the *AUC* value of the DDI strategy (0.97) was substantially higher than that of the others (0.75 and 0.72, respectively), which were similar to the results assessed by *ROC* curves. In conclusion, this finding indicated that classification correctness (assessed by *ACC*, *ROC*, and *AUC*) and prediction stability (evaluated by *MCC*) of DDI strategy were found consistently better compared with those strategies based on single dataset.

### 2.3. Comparative Analysis on the Robustness of the DEGs Identified by Different Analytical Strategies

Apart from prediction capacity simultaneously assessed by classification correctness and prediction stability, the robustness of identified DEGs was widely accepted as an additional key metric with its underlying theory distinct from that of prediction ability [69,74]. So far, *overlap* value had been recognized as the quantitative measure of the robustness of identified DEGs [75]. The higher *overlap* values represented the more robust DEGs identified by a given strategy. Herein, one sub-dataset was first generated by randomly selecting 2/3 of both cases and controls in the studied dataset, and ten iterations of the same procedure generated ten sub-datasets. For each sub-dataset, a list of DEGs were then identified by LMEB method (fold change > 1.5 and adjusted *p*-value < 0.05), and the value of *overlap* between any two sub-datasets was calculated using their corresponding lists of DEGs. In total, there were 45 ($C_{10}^{2}$) *overlap* values denoting all possible combinations between any two sub-datasets. Finally, *overlap* values of three different analytical strategies were compared. As shown in the upper section of Table 3, the total numbers of DEGs identified by ten sub-datasets together with the median values of *overlap* were provided. It was obvious that the total numbers of identified DEGs among ten sub-datasets varied significantly (from 370 to 1310). The median *overlap* of DDI strategy (0.69) was found larger than that of the other strategies (0.64 and 0.42, respectively).

Compared with the median value of *overlap*, the statistical difference of 45 *overlap* values between different analytical strategies was more meaningful to reveal their level of robustness. Therefore, the comprehensive statistical comparison of robustness among strategies was conducted and illustrated in Figure 2D. *Overlap* values of three strategies were colored in blue, green and orange. Apart from the enhanced median value of *overlap* by DDI strategy, all *overlap* values of DDI strategy were found significantly higher (*p*-value < 0.05) than that of the other two. Particularly, as illustrated in Figure 2D, the statistical differences between DDI strategy and others (*p*-value) were all lower than 1.00 × 10^−3^ (1.89 × 10^−24^ between A and C; 5.56 × 10^−5^ between B and C). Moreover, the majority of the *overlap* values of DDI strategy were larger than 0.6, while that of the other strategies were lower than 0.6. This finding indicated that the DDI strategy performed better than others in the robustness of the identified DEGs. Additionally, Table 3 (lower section) provided the numbers and percentages of DEGs simultaneously discovered by N (N ≥ 6, ≥7, ≥8, ≥9, =10) sub-datasets. It was very clearly that the robustness of the DEGs identified by DDI strategy was much better than other two strategies in terms of both the number and percentage of co-identified DEGs. Particularly, about one third of the DEGs of DDI strategy were identified by over five sub-datasets, which was substantially higher than that of other two (28% and 10%, respectively). 17% of the DEGs of DDI strategy could be identified by all sub-datasets, which was still significantly higher than that of other two (14% and 4%, respectively).

### 2.4. Annotating the Immune-Related DEGs and Candidate Target Discovery for PA Immunotherapy

The discussion above testified the effectiveness of the adopted feature selection algorithm and revealed the superior robustness and classification ability of the proposed DDI strategy compared with that based on the single dataset. To maximumly enhance the prediction power of constructed model, the most comprehensive set of PA-related microarray data were collected (Table 1) and integrated to form data **D** (Figure 1). To the best of our knowledge, the dataset **D** was the largest one among current PA-related transcriptomic analyses. Using LMEB method (fold change > 1.5 and adjusted *p*-value < 0.05), a total of 370 DEGs were discovered. As shown in Table 4, the top-ranked DEGs (top-20 for up-regulation & another top-20 for down-regulation) were provided, and the identified DEGs that could not be found by the other two strategies were highlighted in bold font. These top-ranked DEGs and their relevance to PAs were reviewed by comprehensive literature review, and a variety of DEGs were identified as relevant to PAs by experiments. For example, the mRNA expression of *TSHB* was found to be absent in most PA patients [76]. Different from normal pituitary, the *GAL* was reported to be rarely expressed in somatotroph adenoma and prolactinoma [77]. The expression of *GAL* was substantially suppressed in the tissue of PA patients compared with that in healthy pituitary [47]. The *CEL* was identified to regulate the hormone secretion in adenomatous pituitary cells [78]. The high expression of *PRL* and under-expression of *RPS29* were observed in pituitary adenomas [79]. The *c-Fos* overexpression was found to be much less common in pituitary tumors and did not necessarily correlate with the ability of tumor to become invasive [80]. Pathway analysis facilitated the discovery of *PMAIP1* as important in the tumorigenesis and progression of PAs [81]. The serum *AGR2* level was significantly higher in the serum of PA patients than the patients with other sellar lesions, which suggests *AGR2* a potential marker for PAs’ diagnosis [82]. By annotating those 370 DEGs using GO terms, 66 (17.8%) DEGs were discovered as closely immune-related. Table 5 provided a full list of the immune-related DEGs together with their fold changes and *p*-values. Due to their close relationship with human immune system and the differential expression between PA patients and healthy individuals, they are potential target candidates for PA immunotherapy.

**Figure 3:**
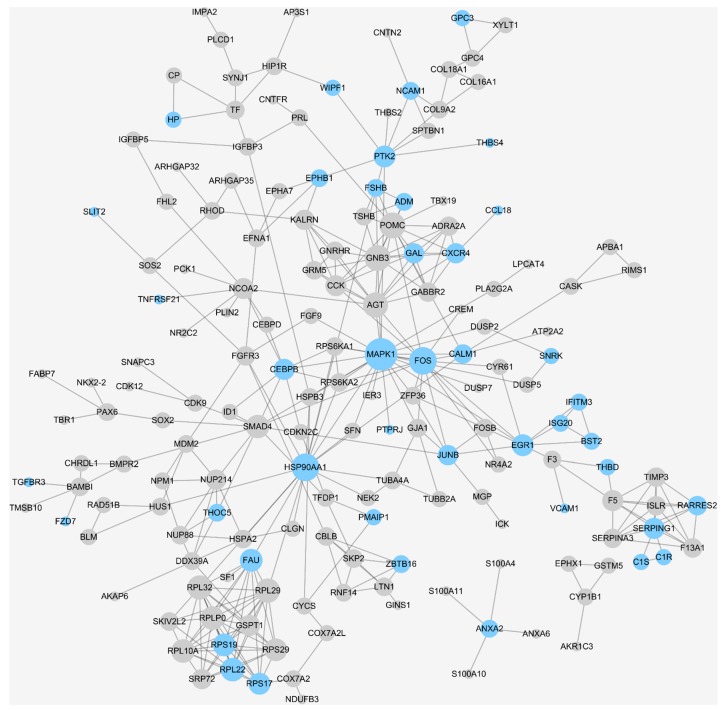
Human protein–protein interaction (PPI) network constructed based on 370 DEGs identified in this study. The PPI information was collected from the 2017 version of the STRING database and visualized using the Cytoscape software package. The diameter of each protein was defined by its network degree, and the proteins colored in blue were identified to be “immune-related” in this study.

The human protein–protein interaction (PPI) network analyses (Figure 3) further revealed relations among the identified DEGs and immune-related ones (highlighted in blue). Among the identified target candidates for PA immunotherapy, *MAPK1*, *FOS* and *HSP90AA1* were the top three immuno-candidates with the highest connectivity (network degree) to other human proteins. Moreover, galanin (*GAL*) was identified as greatly downregulated DEGs in the pituitary glands of PA patients. Due to it immunomodulatory property in PA, the GAL could be expected as a promising target candidate for PA immunotherapy, and this finding was in accordance to previous work [47]. The dysregulation of *LMO4* was identified in this study, which induced *STAT3* activation in brain [83] and subsequently regulated the expression of programmed death-ligand 1 (*PD-L1*) [84]. Since the upregulated expression of immunosuppressive protein *PD-L1* was observed in the pituitary gland of PA patients [43], the inhibition of *LMO4/STAT3/PD-L1* signaling could block the immune evasion by recovering the recognition and elimination of cancer cells via CD8^+^ lymphocytes [48]. Furthermore, the transforming growth factor-beta (*TGFB*) was reported to be one of the chief immune suppressive mediators of PRL secretion and PA proliferation [49,50]. Its receptor, *TGFBR3*, was also discovered in this study as a DEG, which could be another target candidate for developing novel PA immunotherapy.

## 3. Materials and Methods

### 3.1. Construction of Analytical Datasets and Discovery of Differentially Expressed Genes

Pituitary glands were wildly recognized as the major locus of PA’s dysfunction [63]. In this study, a variety of microarray studies based on the tissues of pituitary gland were thus collected through searching “pituitary adenoma”, “pituitary tumor”, “pituitary neoplasm”, “pituitary macroadenoma”, “pituitary carcinoma”, and “pituitary cancer” in the Gene Expression Omnibus (GEO) [85]. As a result, seven independent microarray studies were collected. As shown in Table 1, each dataset comprises a cohort of PA patients and/or another cohort of healthy controls. Among these studies, a limited number of both diseased and healthy samples were reported (~4–16 samples for the PA patients and ~0–9 samples for healthy people). In this study, four analytical datasets were constructed for (1) evaluating the effectiveness of the adopted feature selection algorithm, (2) conducting a performance comparison between the DDI strategy and the strategy of a single dataset, and (3) constructing the final model for identifying the immune-related molecule underlying PA’s development. In particular, the four analytical datasets included (as illustrated in Figure 1 and Table 1): (A) GSE51618, (B) GSE26966, (C) DDI of five datasets, and (D) DDI of seven datasets. Datasets (A–C) were used for validating feature selection method and comparing the performances of DDI strategy and the strategy of single dataset, and dataset D was applied to identify the target candidates for the immunotherapy of PA.

The direct data integration (DDI) strategy combining multiple datasets was carried out in the *R* environment (available online: http://www.r-project.org). The raw data (the CEL file) was first read, log transformed, and normalized, and all parameters were set as default. Then, outliers in each dataset were checked and removed, and all probe sets were mapped to their corresponding genes by *Bioconductor* [86]. Average expression value was retained if one gene was mapped to multiple probes. To remove the batch effect among datasets, Z score transformation [87,88,89] (provided in Equation (1)) was used to adjust the gene expression levels in each dataset.

(1) $$Z\;\mathrm{score}\;=\frac{x_{i}-\bar{x}}{\delta }$$

where $x_{i}$ refers to the raw intensity of each gene, $\bar{x}$ indicates the average intensity of all genes within single experiment and δ represents the standard deviation (SD) of all expression intensities in one array. After this process, the mean Z score for each array became zero with SD equaling one. The DDI strategy was applied to construct the dataset C (integrating GSE22812, GSE26966, GSE4237, GSE46311, and GSE51618) and D (integrating GSE2175, GSE22812, GSE26966, GSE36314, GSE4237, GSE46311, and GSE51618). As a result, the dataset C contained 60 PA patients and 12 healthy people, and dataset D contained 68 PA patients and 16 healthy individuals (Figure 1). The differentially expressed genes (DEGs) between the PA patients and healthy individuals were identified using the linear models and empirical Bayes (LMEB) [90] by the *R* package *limma* [91]. Herein, the genes with fold change > 1.5 and adjusted *p*-value < 0.05 were identified as the differentially expressed genes between case and control samples (Figure 1).

### 3.2. Validation of the Constructed Prediction Models Based on Different Analytical Datasets

A systematic validation and comparison of the prediction models constructed based on three analytical datasets (A–C) was conducted by evaluating the relevance level to PAs of the identified DEGs and by assessing the robustness of marker discovery and the classification ability of the constructed models.

#### 3.2.1. The Level of Relevance of the Identified DEGs to Pituitary Adenoma

For complex disorders like PA, the identified DEGs are expected to contain a substantial percentage of PA-related genes [92], and a certain number of irrelevant genes may be inevitably selected due to measurement variability. Herein, a comprehensive literature review was performed to investigate the PA relevance of the top-ranked DEGs. Moreover, the pathway enrichment analyses on the identified DEGs were also conducted to identify the significantly overrepresented KEGG pathways using hypergeometric tests (adjusted *p*-value < 0.05) [93]. According to another comprehensive literature review on the top-ranked pathways playing key roles in PA, the level of PA relevance of the pathways enriched by three different strategies were compared. Finally, the gene ontology (GO) enrichment analyses using biological process (BP) and molecular function (MF) by the identified DEGs were conducted to identify the significantly overrepresented GO terms using hypergeometric tests (adjusted *p*-value < 0.05) [93]. The level of PA relevance of the top-ranked GO terms was also reviewed, analyzed, and compared among three different strategies.

#### 3.2.2. The Classification Capacity of the Identified DEGs Assessed by the Independent Validation Dataset

The classification abilities of the DEGs identified by three different strategies (Dataset A, B and C shown in Figure 1) were assessed and compared by predicting PA outcomes of the independent validation dataset containing eight PA patients and four healthy controls. To statistically maximize both the prediction power of the constructed models and the validation effectiveness, the independent validation dataset was constructed by two datasets with the smallest amount of case and control samples: GSE2175 (four cases and one control) and GSE36314 (four cases and three controls). The classification models were constructed by the classifier of support vector machine (SVM) [94]. A variety of popular assessment metrics were adopted, which included true positive (*TP*), true negative (*TN*), false positive (*FP*), false negative (*FN*), accuracy (*ACC*), sensitivity (*SEN*), specificity (*SPE*), Matthews correlation coefficient (*MCC*), receiver operating characteristics (*ROC*) curve, and the area under *ROC* curve (*AUC*). All these metrics were frequently used in current Omics researches [95,96,97,98].

#### 3.2.3. The Robustness of the Identified DEGs among Different Sampling Groups

The robustness of the identified DEGs among different samplings groups was widely accepted to be another metric for assessing the constructed prediction models [69]. Particularly, *overlap* value between two sampling groups was applied to quantify the robustness of the identified DEGs [75]. In this study, a sub-dataset was first generated by randomly selecting 2/3 samples of the original dataset, and ten iterations of this selection procedure resulted in ten sub-datasets. For each sub-dataset, a list of DEGs were identified by LMEB (fold change > 1.5 and adjusted *p*-value < 0.05), and the *overlap* value between any two sub-datasets was calculated using their corresponding lists of DEGs identified. In total, there were 45 ($C_{10}^{2}$) *overlap* values denoting all possible combinations between any two sub-datasets. Finally, the *overlap* values of three different strategies were compared to assess their robustness of the identified DEGs.

### 3.3. Annotating the Immune-Related DEGs and Candidate Target Discovery for PA Immunotherapy

Due to the substantially enhanced robustness and classification capacity of the DDI strategy discovered in this study, the DDI of all seven datasets (Table 1) was conducted to achieve the most comprehensive discovery of DEG between patients and healthy individuals. The method applied for data integration and batch effects removal was shown in the first section of Materials and Methods section. The samples integrated in constructing the final prediction model included 68 PA patients and 16 healthy individuals. To the best of our knowledge, this newly integrated dataset is the largest one among the current PA-related transcriptomic analyses. Based on the comprehensive dataset D integrated above (Figure 1), DEGs were further identified using the LMEB method (fold change > 1.5 and adjusted *p*-value < 0.05). Then, GO terms were used to annotate and identify immune-related DEGs. Moreover, the human protein–protein interaction (PPI) network analysis was introduced to explore the connection among identified DEGs [99]. In particular, the STRING database [100] was adopted to the construct human PPI network, and only PPI data of high confidence levels (>0.7) were applied in this study. The identified DEGs together with the STRING human PPI network were input into Cytoscape [101] for visualization, and only the identified DEGs were displayed (round circles). The immune-related DEGs annotated by GO terms were colored by light blue.

## 4. Conclusions

In this study, the direct data integration strategy was proposed, and its classification ability and robustness were assessed in this study. Compared with the traditional strategy based on a single dataset, the DDI strategy was found to greatly enhance the robustness of identified DEGs, which may be attributed to the significant enlargement of the sample size. Based on the comprehensive analysis of the available PA-related transcriptomics data, 370 DEGs were identified, and 66 immune-related DEGs were annotated and proposed as target candidates for PA immunotherapy. Using the PPI network analysis and literature review, several promising candidate targets (*GAL*, *LMO4*, *STAT3*, *PD-L1*, *TGFB* and *TGFBR3*) were further proposed for PA immunotherapy.

## Figures and Tables

**Table 1:** Pituitary adenomas related microarray datasets collected for analysis in this study.

GEO ID	Samples PA:NP *^a^*	Description of the Collected Datasets for Studying on	Microarray Platform	Reference of the Studied Datasets
GSE2175	4:1	GeneChip arrays of 1 healthy and 4 pituitary adenoma samples	HG-U133A	[61]
GSE22812	13:0	Genomic hybridization arrays of 13 pituitary adenoma samples	GE Healthcare	[53]
GSE26966	14:9	GeneChip arrays of 9 healthy and 14 pituitary adenoma samples	HG-U133_Plus_2	[62]
GSE36314	4:3	Genome arrays of 3 healthy and 4 pituitary adenoma samples	HG_U95Av2	[63]
GSE4237	10:0	Affymetrix oligo arrays of 10 pituitary adenoma samples	HG-U133_Plus_2	[52]
GSE46311	16:0	Affymetrix Human Gene arrays of 16 pituitary adenoma samples	HuGene-1_0-st	[51]
GSE51618	7:3	Agilent arrays of 3 healthy and 7 pituitary adenoma samples	Agilent-hgug4112a	[64]

*^a^* PAs: pituitary adenomas; NP: normal pituitary.

**Table 2:** Prediction ability of three classifiers. These classifiers were build using GSE51618, GSE26966 and the combination of GSE22812, GSE26966, GSE4237, GSE46311, and GSE51618. Prediction ability was assessed by the integration of GSE36314 and GSE2175 as the independent validation dataset.

Datasets	TP *^a^*	FN *^a^*	TN *^a^*	FP *^a^*	ACC *^b^*	SEN *^b^*	SPE *^b^*	MCC *^b^*	AUC *^c^*
A: GSE51618	4	4	4	0	0.67	0.50	1.00	0.50	0.75
B: GSE26966	5	3	3	1	0.67	0.63	0.75	0.35	0.72
C: DDI Strategy	7	1	4	0	0.92	0.88	1.00	0.84	0.97

*^a^* TP: true positive; TN: true negative; FP: false positive; FN: false negative. *^b^* ACC: accuracy; SEN: sensitivity; SPE: specificity; MCC: Matthews correlation coefficient. *^c^* AUC: area under the curve.

**Table 3:** The robustness of the identified markers assessed by 10 random sampling datasets.

Dataset		A: GSE51618	B: GSE26966	C: DDI Strategy
Overlap Median across 10 Samplings		0.42	0.64	0.69
No. of DEGs Identified by the *n*th Sampling Dataset (*n*=)	1	230	244	175
	2	149	312	147
	3	179	332	140
	4	319	262	134
	5	284	328	132
	6	633	349	282
	7	361	278	171
	8	283	295	172
	9	377	243	223
	10	585	295	192
No. of DEGs Identified		1310	370	410
No. (Percent) of DEGs Co-discovered by *N* Sampling Datasets	10	56 (0.04)	52 (0.14)	71 (0.17)
	≥9	73 (0.06)	63 (0.17)	90 (0.22)
	≥8	87 (0.07)	75 (0.20)	107 (0.26)
	≥7	103 (0.08)	87 (0.24)	119 (0.29)
	≥6	129 (0.10)	104 (0.28)	133 (0.32)

**Table 4:** Top 20 up- and downregulated DEGs identified by combining all seven datasets in Table 1. These DEGs that could not be identified by both GSE62966 and GSE51618 dataset were highlighted in bold font.

No.	Entrez	Symbol	LogFC	*p*-Value	GES62966	GSE51618
1	5443	*POMC*	−3.85	2.79 × 10^−18^	−8.00	−7.09
2	7252	*TSHB*	−3.26	6.65 × 10^−16^	−5.96	−8.07
3	51,083	*GAL*	−2.69	4.22 × 10^−21^	−5.10	−8.03
4	1056	*CEL*	−2.22	9.21× 10^−10^	−5.73	−2.23
5	3240	*HP*	−2.21	1.06 × 10^−9^	−4.10	-
6	3397	*ID1*	−2.08	3.85 × 10^−16^	−3.10	−3.84
7	5446	*PON3*	−2.00	2.01 × 10^−7^	−3.46	-
8	5617	*PRL*	−1.93	6.41 × 10^−3^	−7.33	-
9	1410	*CRYAB*	−1.88	1.31 × 10^−12^	−2.57	-
10	4885	*NPTX2*	−1.87	8.75 × 10^−9^	−4.18	-
**11**	**6161**	***RPL32***	**−1.83**	**1.05 × 10^−2^**	-	-
12	4821	*NKX2-2*	−1.82	3.36 × 10^−7^	−6.26	−0.83
13	2697	*GJA1*	−1.64	5.94 × 10^−8^	−3.73	-
14	5105	*PCK1*	−1.61	1.74 × 10^−5^	−6.43	−2.38
**15**	**6235**	***RPS29***	**−1.54**	**1.18 × 10^−2^**	-	-
16	12	*SERPINA3*	−1.48	4.62 × 10^−4^	−4.15	-
17	2353	*FOS*	−1.45	2.85 × 10^−7^	−3.80	-
18	5366	*PMAIP1*	−1.44	4.11 × 10^−7^	−3.85	−2.39
**19**	**6146**	***RPL22***	**−1.42**	**2.91 × 10^−2^**	-	-
20	10,551	*AGR2*	−1.41	5.24 × 10^−5^	−2.47	−3.75
**21**	**22,999**	***RIMS1***	**0.78**	**3.05 × 10^−2^**	-	-
22	8573	*CASK*	0.78	5.10 × 10^−6^	0.85	1.48
**23**	**5795**	***PTPRJ***	**0.78**	**1.01 × 10^−2^**	-	-
24	1272	*CNTN1*	0.80	7.88 × 10^−5^	1.70	-
**25**	**22,858**	***ICK***	**0.81**	**1.53 × 10^−2^**	-	-
**26**	**23,390**	***ZDHHC17***	**0.81**	**4.94 × 10^−3^**	-	-
**27**	**51,755**	***CDK12***	**0.81**	**3.45 × 10^−2^**	-	-
28	4684	*NCAM1*	0.81	5.74 × 10^−3^	0.74	-
**29**	**4863**	***NPAT***	**0.81**	**1.07 × 10^−2^**	-	-
30	57,125	*PLXDC1*	0.82	6.76 × 10^−4^	0.76	-
31	9472	*AKAP6*	0.83	1.75 × 10^−3^	0.86	-
**32**	**23,101**	***MCF2L2***	**0.83**	**2.02 × 10^−2^**	-	-
**33**	**868**	***CBLB***	**0.84**	**3.45 × 10^−2^**	-	-
34	8490	*RGS5*	0.84	3.25 × 10^−4^	1.41	-
35	1006	*CDH8*	0.85	2.18 × 10^−3^	2.94	-
**36**	**55,752**	***SEPT11***	**0.87**	**1.13 × 10^−2^**	-	-
37	5149	*PDE6H*	0.89	1.67 × 10^−2^	1.36	-
38	1641	*DCX*	0.91	6.76 × 10^−4^	3.14	-
39	29,899	*GPSM2*	0.91	1.73 × 10^−2^	1.04	-
40	23,305	*ACSL6*	0.91	3.51 × 10^−3^	0.90	-

**Table 5:** Immune-related DEGs identified in this study with fold change, *p*-value, and their representative biological processes and molecular functions.

Entrez ID	Gene Symbol	LogFC	*p*-Value	Representative GO Biological Processes and Molecular Functions
51,083	*GAL*	−2.69	4.22 × 10^−21^	negative regulation of immune system process; regulation of immune process
3240	*HP*	−2.21	1.06 × 10^−9^	immune system process
2353	*FOS*	−1.45	2.85 × 10^−7^	positive regulation of immune system process; regulation of immune response
5366	*PMAIP1*	−1.44	4.11 × 10^−7^	immune effector process; immune system process
6146	*RPL22*	−1.42	2.91 × 10^−2^	immune system development; immune system process
7060	*THBS4*	−1.33	7.19 × 10^−6^	positive regulation of immune system process; regulation of immune process
10,410	*IFITM3*	−1.28	8.39 × 10^−6^	innate immune response; immune effector process; immune system process
2488	*FSHB*	−1.28	5.95 × 10^−3^	regulation of immune system process
133	*ADM*	−1.28	4.34 × 10^−7^	humoral immune response; immune response; immune system process
2719	*GPC3*	−1.22	3.09 × 10^−9^	immune system development; immune system process
7852	*CXCR4*	−1.22	3.24 × 10^−9^	immune system process
347	*APOD*	−1.21	2.78 × 10^−5^	negative regulation of immune system process; regulation of immune process
9353	*SLIT2*	−1.21	6.81 × 10^−10^	negative regulation of immune system process; regulation of immune process
8324	*FZD7*	−1.21	1.74 × 10^−8^	immune system development; immune system process
2197	*FAU*	−1.19	1.35 × 10^−2^	humoral immune response; innate immune response in mucosa
4783	*NFIL3*	−1.14	8.44 × 10^−5^	immune response; immune system process
6218	*RPS17*	−1.13	2.70 × 10^−2^	immune system process
5950	*RBP4*	−1.08	1.79 × 10^−2^	positive regulation of production of molecular mediator of immune response
5919	*RARRES2*	−1.06	4.34 × 10^−7^	positive regulation of immune system process; regulation of immune process
3669	*ISG20*	−1.03	8.23 × 10^−8^	innate immune response; immune effector process; immune system process
3059	*HCLS1*	−1.02	6.70 × 10^−5^	positive regulation of immune system process; regulation of immune process
6279	*S100A8*	−1.01	1.99 × 10^−3^	innate immune response; immune response; immune system process
710	*SERPING1*	−1.00	6.70 × 10^−6^	adaptive immune response; b cell mediated immunity; humoral immune response
716	*C1S*	−1.00	5.90 × 10^−4^	adaptive/innate immune response; Leukocyte mediated immunity
7056	*THBD*	−1.00	2.52 × 10^−6^	immune system process
715	*C1R*	−0.98	3.31 × 10^−6^	adaptive/innate immune response; positive regulation of immune system process
1672	*DEFB1*	−0.92	1.44 × 10^−4^	humoral immune response; innate immune response in mucosa
2669	*GEM*	−0.89	2.22 × 10^−4^	immune response; immune system process
1958	*EGR1*	−0.86	2.45 × 10^−3^	innate immune response; immune response; immune system development
6662	*SOX9*	−0.86	3.43 × 10^−4^	negative regulation of immune system process; regulation of immune process
3426	*CFI*	−0.84	6.05 × 10^−10^	adaptive immune response; leukocyte/lymphocyte mediated immunity
7412	*VCAM1*	−0.84	6.87 × 10^−7^	innate immune response; positive regulation of immune system process
1366	*CLDN7*	−0.84	4.27 × 10^−3^	regulation of immune effector process; regulation of immune system process
3726	*JUNB*	−0.84	2.43 × 10^−5^	immune system development; immune system process
9796	*PHYHIP*	−0.82	2.78 × 10^−3^	regulation of immune effector process; regulation of immune system process
7049	*TGFBR3*	−0.81	3.58 × 10^−3^	immune response; immune system development; immune system process
3958	*LGALS3*	−0.80	4.63 × 10^−2^	innate immune response; negative regulation of immune effector process
3485	*IGFBP2*	−0.78	1.23 × 10^−3^	positive regulation of immune system process; regulation of immune process
1051	*CEBPB*	−0.75	2.38 × 10^−3^	negative regulation of immune system process; regulation of immune process
302	*ANXA2*	−0.72	1.55 × 10^−2^	immune system development; immune system process
5806	*PTX3*	−0.72	2.46 × 10^−3^	innate immune response; immune effector process; immune system process
3320	*HSP90AA1*	−0.71	1.90 × 10^−2^	positive regulation of immune system process; activation of immune response
7704	*ZBTB16*	−0.69	3.81 × 10^−2^	negative/positive regulation of immune system process
8543	*LMO4*	−0.69	1.35 × 10^−2^	immune system development; immune system process
6223	*RPS19*	−0.69	1.25 × 10^−2^	regulation of innate immune response; immune system development
4057	*LTF*	−0.68	3.41 × 10^−2^	humoral immune response; innate immune response in mucosa
100,133,941	*CD24*	−0.65	5.68 × 10^−3^	positive regulation of immune system process; immune system process
684	*BST2*	−0.64	8.65 × 10^−3^	humoral/innate immune response; negative regulation of immune response
23,543	*RBFOX2*	0.59	5.42 × 10^−3^	regulation of immune system process
5747	*PTK2*	0.59	1.12 × 10^−2^	activation of immune response; positive regulation of immune response
2047	*EPHB1*	0.60	8.01 × 10^−3^	immune system process; immunological synapse formation
27,242	*TNFRSF21*	0.60	4.66 × 10^−3^	adaptive/humoral immune response; negative regulation of immune process
8754	*ADAM9*	0.61	1.68 × 10^−2^	immune system process
3175	*ONECUT1*	0.64	1.36 × 10^−3^	immune system development; immune system process
54,861	*SNRK*	0.64	1.61 × 10^−2^	immune system development; immune system process
801	*CALM1*	0.64	6.76 × 10^−4^	immune response regulating cell surface receptor signaling pathway
51,752	*ERAP1*	0.65	2.84 × 10^−2^	regulation of innate immune response; regulation of immune system process
7456	*WIPF1*	0.65	1.59 × 10^−3^	activation of immune response; immune system process
4982	*TNFRSF11B*	0.66	3.67 × 10^−2^	immune response; immune system process
6362	*CCL18*	0.67	3.72 × 10^−2^	innate immune response; immune response; immune system process
30,849	*PIK3R4*	0.67	2.02 × 10^−2^	activation/positive regulation of innate immune response
5594	*MAPK1*	0.71	2.18 × 10^−3^	regulation of immune system process; immune system development
8563	*THOC5*	0.71	7.07 × 10^−3^	immune system development; immune system process
8473	*OGT*	0.72	1.16 × 10^−2^	positive regulation of immune system process
5795	*PTPRJ*	0.78	1.01 × 10^−2^	negative/positive regulation of immune system process
4684	*NCAM1*	0.81	5.74 × 10^−3^	innate immune response; immune response; immune system process

## Supplementary Materials

The following are available online at http://www.mdpi.com/1422-0067/20/1/151/s1.
