# Supplementary material for: Biomarker Discovery for Immunotherapy of Pituitary Adenomas: Enhanced Robustness and Prediction Ability by Modern Computational Tools
Source: Int J Mol Sci. 2019 Jan 3;20(1):151. doi: 10.3390/ijms20010151 (PMC6337483; doi:10.3390/ijms20010151)
Supplement: Supplementary file 1 [file ijms-20-00151-s001.pdf]

Supplementary Information for:

Biomarker Discovery for Immunotherapy of Pituitary Adenomas: Enhanced Robustness and Prediction Ability by Modern Computational Tools

Qingxia YANG[1,2], Yunxia WANG[2], Song ZHANG[2], Jing TANG[1,2], Fengcheng LI[2], Jiayi YIN[2], Yi LI[2], Jianbo FU[2], Bo LI[1], Yongchao LUO[2], Weiwei XUE[1,*] and Feng ZHU[1,2,*]

[1] Innovative Drug Research and Bioinformatics Group, School of Pharmaceutical Sciences, Chongqing University, Chongqing 401331, China

[2] Innovative Drug Research and Bioinformatics Group, College of Pharmaceutical Sciences, Zhejiang University, Hangzhou 310058, China

* Corresponding author: Prof. Feng ZHU (zhufeng@zju.edu.cn; prof.zhufeng@gmail.com)

Supplementary Table 1. Relevance between PA and the top-ranked (top-5 up & another top-5 down regulated) DEGs identified in this study confirmed by published literatures. N.A.: not available.

Gene Entrez	Gene Symbol	LogFC	*p*-value	Relevance between the Identified DEGs and PA Confirmed by the Comprehensive Literature Reviews
**A: Strategy of Single Dataset (GSE51618)**				
4435	CITED1	2.97	8.79E-03	N.A.
1191	CLU	2.91	1.13E-02	CLU expression in pituitary adenomas was found significantly higher than in non-neoplastic adenohypophyses[1]
1442	CSH1	-10.29	1.72E-06	N.A.
1443	CSH2	-11.09	4.12E-07	N.A.
1444	CSHL1	-10.39	4.13E-08	N.A.
8788	DLK1	-9.29	4.15E-05	DLK1 expression was reduced 2500-fold in nonfunctioning pituitary adenomas, compared with normal pituitary[2]
56884	FSTL5	3.25	1.23E-02	N.A.
2688	GH1	-11.17	1.71E-06	GH-immunoreactivity was detected in most adenoma cells in the cases of unusual chromophobic adenomas[3]
3006	HIST1H1C	2.87	4.06E-02	N.A.
140679	SLC32A1	3.20	3.59E-04	N.A.
**B: Strategy of Single Dataset (GSE26966)**				
158763	ARHGAP36	-7.60	2.21E-09	N.A.
339479	BRINP3	4.84	1.20E-05	BRINP3 was overexpressed in pituitary adenomas, compared with the normal people[4]
389073	C2orf80	4.81	2.12E-04	N.A.
1442	CSH1	-8.28	3.15E-25	N.A.

79695	GALNT12	4.08	3.59E-07	N.A.
2688	GH1	-9.71	1.46E-16	It was showed a faint GH1-immunoreactivity in pituitary adenomas[3]
5443	POMC	-8.00	1.00E-17	In silent subtype 2 and 3 pituitary adenomas, POMC had a diffuse low level or was absent, compared to the normal[5]
5617	PRL	-7.33	1.49E-07	PRL gene expression was rare pituitary adenomas[6]
201780	SLC10A4	4.17	7.53E-06	N.A.
91752	ZNF804A	4.91	3.36E-09	N.A.
**C: DDI Strategy Integrating Five Datasets (GSE22812, GSE26966, GSE4237, GSE46311 and GSE51618)**				
23305	ACSL6	0.91	3.51E-03	N.A.
1056	CEL	-2.22	9.21E-10	CEL expression was lacking in PRL cells pituitary adenomas, which is different from the normal pituitary[7]
1641	DCX	0.91	6.76E-04	DCX expression was positively correlated with N-cadherin, which was increased in pituitary adenomas[8,9]
51083	GAL	-2.69	4.22E-21	Different from normal pituitary, GAL was rarely expressed in somatotroph adenomas and prolactinomas[10]
29899	GPSM2	0.91	1.73E-02	PA pathogenesis is regulated by PI3K/AKT, which is the key pathway activated by GPSM2 during tumor growth[11,12]
3240	HP	-2.21	1.06E-09	The levels of five isoforms of HP were significantly decreased after GH substitution in PA patients[13]
5149	PDE6H	0.89	1.67E-02	N.A.
5443	POMC	-3.85	2.79E-18	In silent subtype 2 and 3 pituitary adenomas, POMC had a diffuse low level or was absent, compared to the normal[5]
55752	SEPT11	0.87	1.13E-02	N.A.
7252	TSHB	-3.26	6.65E-16	The mRNA expression of TSHB was absent in most PA patients[14]

Supplementary Table 2. Relevance between PA and the top-10 KEGG pathways identified here confirmed by published literatures. N.A.: not available.

KEGG Pathway	*p*-value	Relevance between SCZ and the Enriched Pathways Confirmed by the Comprehensive Literature Reviews
**A: Strategy of Single Dataset (GSE51618)**		
Alzheimer's disease	2.00E-12	N.A.
Oxidative phosphorylation	1.96E-09	Oxidative phosphorylation is revealed as the significant pathway in human pituitary adenoma by DEP data[15]
Huntington's disease	8.43E-09	N.A.
Phosphatidylinositol signaling system	1.96E-08	Phosphatidylinositol signaling system was found to be disturbed in pituitary tumors[16]
Oocyte meiosis	4.60E-08	Differentially expression genes in pituitary gonadotroph adenomas were enriched in oocyte meiosis pathway[17]
Parkinson's disease	9.81E-08	N.A.
Citrate cycle/TCA cycle	9.81E-08	N.A.
Pathways in cancer	1.54E-07	N.A.
Axon guidance	2.34E-07	N.A.
Focal adhesion	3.39E-07	N.A.
**B: Strategy of Single Dataset (GSE26966)**		
MAPK signaling pathway	2.57E-10	MAPK-signaling abnormality was demonstrated to be significantly associated with a pituitary adenoma[15]
Pathways in cancer	3.74E-10	N.A.
Alzheimer's disease	2.74E-08	N.A.
Axon guidance	3.69E-07	N.A.

DNA replication	5.26E-07	N.A.
Cardiac muscle contraction	5.26E-07	Cardiac muscle contraction is found to be significantly enriched pathway in pituitary adenoma[18]
Endocytosis	1.32E-06	Among pituitary adenoma, clathrin- and caveolar-mediated endocytosis were statistically significant pathways[19]
Huntington's disease	1.51E-06	N.A.
Cell cycle	2.22E-06	Pathogenetic mechanisms of pituitary tumorigenesis is genetic or epigenetic and result in cell cycle dysregulation[20]
Adipocytokine signaling pathway	2.22E-06	N.A.
**C: DDI Strategy Integrating Five Datasets (GSE22812, GSE26966, GSE4237, GSE46311 and GSE51618)**		
TGF-beta signaling pathway	2.83E-06	TGF-beta signaling pathway was significantly enriched for the genes of plurihormonal pituitary adenomas[21]
Ribosome	2.83E-06	Genes encoding ribosomal proteins were under-expressed in GH-secreting pituitary adenomas[22]
MAPK signaling pathway	2.83E-06	MAPK-signaling abnormality was significantly associated with pituitary adenoma[15]
Complement and coagulation cascades	4.15E-06	Genes in complement and coagulation cascades were upregulated in high MGMT expressing pituitary adenomas[23]
Pathways in cancer	2.08E-05	N.A.
Cell cycle	3.28E-05	Pathogenetic mechanisms of pituitary tumorigenesis is genetic or epigenetic and result in cell cycle dysregulation[20]
P53 signaling pathway	3.87E-05	P53 signaling pathway plays an important role in tumorigenesis and progression of plurihormonal pituitary adenomas[21]
Axon guidance	2.49E-04	N.A.
PPAR signaling pathway	4.23E-04	N.A.
Leukocyte transendothelial migration	9.45E-04	Leukocyte transendothelial migration was found to be an important pathway in pituitary adenomas[24]

Supplementary Table 3. Relevance between PA and the top-10 GO biological processes (BPs) identified here confirmed by literatures. N.A.: not available.

GO Biological Process	*p*-value	Relevance between PA and the Enriched GO BPs Confirmed by the Comprehensive Literature Reviews
**A: Strategy of Single Dataset (GSE51618)**		
Small molecule metabolic process	9.39E-41	N.A.
Neurogenesis	2.53E-37	N.A.
Intracellular signal transduction	4.72E-36	Raf/MEK/ERK signalling has been considered to be one of the major and central intracellular signal transduction pathways in pituitary adenomas aetiology[25]
Protein complex subunit organization	4.72E-36	Interleukin 4 receptor complex (including IL-4Ralpha, IL-13Ralpha1 and IL-2Rgammac chains) was overexpressed in invasive pituitary adenomas [26]
Regulation of protein modification process	4.72E-36	DNMT3b, which generated 5-methylcytosine by adding a methyl group directly to a cytosine base, was upregulated in PAs via histone modification[27]
Regulation of transport	2.71E-35	The miRNAs microarray results of GO analysis indicated positive regulation of sodium ion transport [28]
Phosphate containing compound metabolic process	2.47E-34	N.A.
Positive regulation of molecular function	3.23E-34	N.A.
Response to endogenous stimulus	6.87E-34	The cellular Ca2+ transport was associated with stimulus-secretion coupling in prolactin producing rat pituitary adenoma cells[29]
Regulation of phosphorus metabolic process	2.59E-33	N.A.
**B: Strategy of Single Dataset (GSE26966)**		
Regulation of transport	7.33E-74	The miRNAs microarray results of GO analysis indicated positive regulation of sodium ion transport[28]
Neurogenesis	7.9E-73	N.A.
Cellular response to organic substance	1.62E-71	The results of the GO analysis from normal pituitary and PA tissues indicated the functions of the downregulated genes were associated with the response to organic substance [30]
Response to oxygen containing compound	2.47E-70	N.A.

Regulation of multicellular organismal development	2.63E-69	N.A.
Regulation of cell differentiation	3.3E-69	Epigenetic chromatin structures were involved in regulation of the differentiation of pituitary adenomas [31]
Intracellular signal transduction	4.11E-69	The Raf/MEK/ERK signaling has been considered to be one of the major and central intracellular signal transduction pathways in pituitary adenomas aetiology [25]
Tissue development	6.09E-67	N.A.
Small molecule metabolic process	2.29E-66	N.A.
Response to endogenous stimulus	8.85E-66	The cellular Ca2+ transport was associated with stimulus-secretion coupling in prolactin producing rat pituitary adenoma cells [29]
**C: DDI Strategy Integrating Five Datasets (GSE22812, GSE26966, GSE4237, GSE46311 and GSE51618)**		
Regulation of cell proliferation	2.72E-38	Immunostaining assay indicated AIB1 participated in the regulation of proliferation of tumoral cells in prolactinomas[32]
Response to endogenous stimulus	2.62E-31	The cellular Ca2+ transport was associated with stimulus-secretion coupling in prolactin producing rat pituitary adenoma cells[29]
Response to external stimulus	5.24E-31	In vitro leptin stimulation of pituitary tumors caused stimulation of FSH and alpha-subunit secretion from a non-functioning adenoma and TSH secretion from a somatotroph adenoma [33]
Tissue development	1.2E-28	N.A.
Positive regulation of response to stimulus	3.64E-27	The results of the GO analysis from blood samples from nine patients with PAs and seven healthy controls indicated the positive regulation of response to stimulus [34]
Positive regulation of cell communication	6.54E-27	Interaction of TGF-beta and b-FGF facilitated the communication between lactotropes and folliculo-stellate cells in prolactinoma formation [35]
Regulation of cell death	2.57E-26	N.A.
Cellular response to organic substance	1.84E-25	The results of the GO analysis from normal pituitary and PA tissues indicated the functions of the downregulated genes were associated with the response to organic substance [30]
Response to oxygen containing compound	1.84E-25	N.A.
Regulation of cell differentiation	2.49E-25	Epigenetic chromatin structures were involved in regulation of the differentiation of pituitary adenomas[31]

Supplementary Table 4. Relevance between PA and the top-10 GO molecular functions (MFs) identified here confirmed by literatures. N.A.: not available.

GO Molecular Function	*p*-value	Relevance between PA and the Enriched GO MFs Confirmed by the Comprehensive Literature Reviews
**A: Strategy of Single Dataset (GSE51618)**		
Enzyme binding	2.26E-37	N.A.
Protein dimerization activity	1.48E-34	N.A.
Macromolecular complex binding	3.48E-34	It has been shown that L-triiodothyronine can be bound with a macromolecular complex in pituitary tumor cells[36]
Identical protein binding	3.67E-30	N.A.
Receptor binding	1.36E-29	The research of nonfunctioning pituitary adenomas showed epidepride was specifically bound with D2 receptor[37]
Ribonucleotide binding	1.06E-28	The significantly enriched GO term for molecular functions of DEGs between PAs and normal control tissues was guanyl ribonucleotide binding[18]
Transporter activity	2.90E-22	N.A.
Transmembrane transporter activity	4.62E-22	N.A.
Molecular function regulator	1.10E-21	mTOR pathway regulators was significantly correlated with the invasion, staging, and tumor growth of pituitary adenomas[38]
Adenyl nucleotide binding	2.78E-21	N.A.
**B: Strategy of Single Dataset (GSE26966)**		
Receptor binding	1.15E-53	The research of nonfunctioning pituitary adenomas showed epidepride was specifically bound with D2 receptor [37]
Enzyme binding	1.15E-53	N.A.
Transporter activity	6.52E-46	N.A.
Protein dimerization activity	4.58E-45	N.A.

Molecular function regulator	3.07E-42	mTOR pathway regulators was significantly correlated with the invasion, staging, and tumor growth of pituitary adenomas[38]
Macromolecular complex binding	7.40E-42	It has been shown that L-triiodothyronine can be bound with a macromolecular complex in pituitary tumor cells[36]
Ribonucleotide binding	8.78E-42	The significantly enriched GO term for molecular functions of DEGs between PAs and normal control tissues was guanyl ribonucleotide binding [18]
Transmembrane transporter activity	1.67E-40	N.A.
Identical protein binding	1.20E-38	N.A.
Lipid binding	1.17E-33	N.A.
**C: DDI Strategy Integrating Five Datasets (GSE22812, GSE26966, GSE4237, GSE46311 and GSE51618)**		
Receptor binding	9.55E-20	The research of nonfunctioning pituitary adenomas showed epidepride was specificly bound with D2 receptor [37]
Identical protein binding	4.58E-14	N.A.
Macromolecular complex binding	1.19E-12	It has been shown that L-triiodothyronine can be bound with a macromolecular complex in pituitary tumor cells[36]
Molecular function regulator	1.22E-12	mTOR pathway regulators was significantly correlated with the invasion, staging, and tumor growth of pituitary adenomas[38]
Ribonucleotide binding	1.22E-12	The significantly enriched GO term for molecular functions of DEGs between PAs and normal control tissues was guanyl ribonucleotide binding[18]
Sulfur compound binding	1.11E-11	The compounds with polar sulfur functions retained the high binding affinity for the calf uterine estrogen receptor in pituitary tumor cells[39,40]
Structural molecule activity	1.11E-11	N.A.
Nucleic acid binding transcription factor activity	2.14E-11	N.A.
Heparin binding	2.14E-11	Heparin-binding secretory transforming gene overexpression in rat pituitary cells mediated lactotroph tumor growth and stimulates PRL transcription [41].
Transcription factor binding	2.14E-11	N.A.

References:

1 A. I. Ekici; B. Eren; N. Turkmen; N. Comunoglu; R. Fedakar. Clusterin expression in non-neoplastic adenohypophyses and pituitary adenomas: cytoplasmic clusterin localization in adenohypophysis is related to aging. Endocr Pathol. 2008, 19(1): 47-53

2 P. Cheunsuchon; Y. Zhou; X. Zhang; H. Lee; W. Chen; Y. Nakayama; K. A. Rice; E. Tessa Hedley-Whyte; B. Swearingen; A. Klibanski. Silencing of the imprinted DLK1-MEG3 locus in human clinically nonfunctioning pituitary adenomas. Am J Pathol. 2011, 179(4): 2120-30

3 A. Yoneda; T. Sano; S. Yamada; A. Obari; Z. R. Qian; E. L. Wang; N. Inosita; E. Kudo. Pituitary adenomas that show a faint GH-immunoreactivity but lack fibrous body: Pit-1 adenoma with endocrinologically low activity. Endocr Pathol. 2010, 21(1): 40-7

4 L. Shorts-Cary; M. Xu; J. Ertel; B. K. Kleinschmidt-Demasters; K. Lillehei; I. Matsuoka; S. Nielsen-Preiss; M. E. Wierman. Bone morphogenetic protein and retinoic acid-inducible neural specific protein-3 is expressed in gonadotrope cell pituitary adenomas and induces proliferation, migration, and invasion. Endocrinology. 2007, 148(3): 967-75

5 L. Stefaneanu; K. Kovacs; E. Horvath; R. V. Lloyd. In situ hybridization study of pro-opiomelanocortin (POMC) gene expression in human pituitary corticotrophs and their adenomas. Virchows Arch A Pathol Anat Histopathol. 1991, 419(2): 107-13

6 E. Baz; W. Saeger; H. Uhlig; S. Fehr; D. K. Ludecke. HGH, PRL and beta HCG/beta LH gene expression in clinically inactive pituitary adenomas detected by in situ hybridization. Virchows Arch A Pathol Anat Histopathol. 1991, 418(5): 405-10

7 S. La Rosa; D. Vigetti; C. Placidi; G. Finzi; S. Uccella; M. Clerici; B. Bartolini; I. Carnevali; M. Losa; C. Capella. Localization of carboxyl ester lipase in human pituitary gland and pituitary adenomas. J Histochem Cytochem. 2010, 58(10): 881-9

8 T. Ide; K. Uchida; K. Suzuki; Y. Kagawa; H. Nakayama. Expression of cell adhesion molecules and doublecortin in canine anaplastic meningiomas. Vet Pathol. 2011, 48(1): 292-301

9 K. Zhou; H. Jin; Y. Luo. Expression and significance of E-cadherin and beta-catenins in pituitary adenoma. Int J Surg Pathol. 2013, 21(4): 363-7

10 B. Leung; T. P. Iisma; K. C. Leung; Y. J. Hort; J. Turner; J. P. Sheehy; K. K. Ho. Galanin in human pituitary adenomas: frequency and clinical significance. Clin Endocrinol (Oxf). 2002, 56(3): 397-403

11 X. Q. He; Y. F. Zhang; J. J. Yu; Y. Y. Gan; N. N. Han; M. X. Zhang; W. Ge; J. J. Deng; Y. F. Zheng; X. M. Xu. High expression of G-protein signaling modulator 2 in hepatocellular carcinoma facilitates tumor growth and metastasis by activating the PI3K/AKT signaling pathway. Tumour Biol. 2017, 39(3): 1010428317695971

12 E. Monsalves; K. Juraschka; T. Tateno; S. Agnihotri; S. L. Asa; S. Ezzat; G. Zadeh. The PI3K/AKT/mTOR pathway in the pathophysiology and treatment of pituitary adenomas. Endocr Relat Cancer. 2014, 21(4): R331-44

13 D. Cruz-Topete; J. O. Jorgensen; B. Christensen; L. Sackmann-Sala; T. Krusenstjerna-Hafstrom; A. Jara; S. Okada; J. J. Kopchick. Identification of new biomarkers of low-dose GH replacement therapy in GH-deficient patients. J Clin Endocrinol Metab. 2011, 96(7): 2089-97

14 J. L. Jameson; A. Klibanski; P. M. Black; N. T. Zervas; C. M. Lindell; D. W. Hsu; E. C. Ridgway; J. F. Habener. Glycoprotein hormone genes are expressed in clinically nonfunctioning pituitary adenomas. J Clin Invest. 1987, 80(5): 1472-8

15 X. Zhan; D. M. Desiderio. Signaling pathway networks mined from human pituitary adenoma proteomics data. BMC Med Genomics. 2010, 313

16 T. Cai; J. Xiao; Z. F. Wang; Q. Liu; H. Wu; Y. Z. Qiu. Identification of differentially coexpressed genes in gonadotrope tumors and normal pituitary using bioinformatics methods. Pathol Oncol Res. 2014, 20(2): 375-80

17 Z. Hou; J. Yang; G. Wang; C. Wang; H. Zhang. Bioinformatic analysis of gene expression profiles of pituitary gonadotroph adenomas. Oncol Lett. 2018, 15(2): 1655-63

18 P. Zhao; W. Hu; H. Wang; S. Yu; C. Li; J. Bai; S. Gui; Y. Zhang. Identification of differentially expressed genes in pituitary adenomas by integrating analysis of microarray data. Int J Endocrinol. 2015, 2015164087

19 X. Zhan; X. Wang; D. M. Desiderio. Pituitary adenoma nitroproteomics: current status and perspectives. Oxid Med Cell Longev. 2013, 2013580710

20 S. Melmed. Pathogenesis of pituitary tumors. Nat Rev Endocrinol. 2011, 7(5): 257-66

21 Z. Jiang; S. Gui; Y. Zhang. Analysis of differential gene expression in plurihormonal pituitary adenomas using bead-based fiber-optic arrays. J Neurooncol. 2012, 108(3): 341-8

22D. S. de Lima; C. S. Martins; B. M. Paixao; F. C. Amaral; L. M. Colli; F. P. Saggioro; L. Neder; H. R. Machado; A. R. dos Santos; D. G. Pinheiro; A. C. Moreira; W. A. Silva, Jr.; M. Castro. SAGE analysis highlights the putative role of underexpression of ribosomal proteins in GH-secreting pituitary adenomas. Eur J Endocrinol. 2012, 167(6): 759-68

23 A. McCormack; W. Kaplan; A. J. Gill; N. Little; R. Cook; B. Robinson; R. Clifton-Bligh. MGMT expression and pituitary tumours: relationship to tumour biology. Pituitary. 2013, 16(2): 208-19

24 C. Cao; W. Wang; C. Ma; P. Jiang. Computational analysis identifies invasion-associated genes in pituitary adenomas. Mol Med Rep. 2015, 12(2): 1977-82

25 Z. Suojun; W. Feng; G. Dongsheng; L. Ting. Targeting Raf/MEK/ERK pathway in pituitary adenomas. Eur J Cancer. 2012, 48(3): 389-95

26 L. Chen; Y. Liu; Y. Hou; Y. Kato; H. Sano; T. Kanno. Expression and structure of interleukin 4 receptor (IL-4R) complex in human invasive pituitary adenomas. Neurosci Lett. 2007, 417(1): 30-5

27 M. Pease; C. Ling; W. J. Mack; K. Wang; G. Zada. The role of epigenetic modification in tumorigenesis and progression of pituitary adenomas: a systematic review of the literature. PLoS One. 2013, 8(12): e82619

28 S. Wu; Y. Gu; Y. Huang; T. C. Wong; H. Ding; T. Liu; Y. Zhang; X. Zhang. Novel Biomarkers for Non-functioning Invasive Pituitary Adenomas were Identified by Using Analysis of microRNAs Expression Profile. Biochem Genet. 2017, 55(3): 253-67

29 J. G. Iversen; K. Sletholt; E. Haug; K. M. Gautvik. On the functional relationship between 45Ca2+ release and prolactin secretion in cultured rat pituitary tumour (GH3) cells. Acta Physiol Scand. 1986, 127(2): 249-55

30 W. Wang; Z. Xu; L. Fu; W. Liu; X. Li. Pathogenesis analysis of pituitary adenoma based on gene expression profiling. Oncol Lett. 2014, 8(6): 2423-30

31 T. Minematsu; S. Miyai; H. Kajiya; M. Suzuki; N. Sanno; S. Takekoshi; A. Teramoto; R. Y. Osamura. Recent progress in studies of pituitary tumor pathogenesis. Endocrine. 2005, 28(1): 37-41

32 J. Carretero; E. J. Blanco; M. Carretero; M. Carretero-Hernandez; M. J. Garcia-Barrado; M. C. Iglesias-Osma; D. J. Burks; J. Font de Mora. The expression of AIB1 correlates with cellular proliferation in human prolactinomas. Ann Anat. 2013, 195(3): 253-9

33 M. Korbonits; M. M. Chitnis; M. Gueorguiev; D. Norman; N. Rosenfelder; M. Suliman; T. H. Jones; K. Noonan; A. Fabbri; G. M. Besser; J. M. Burrin; A. B. Grossman. The release of leptin and its effect on hormone release from human pituitary adenomas. Clin Endocrinol (Oxf). 2001, 54(6): 781-9

34 W. Zhou; C. X. Ma; Y. Z. Xing; Z. Y. Yan. Identification of candidate target genes of pituitary adenomas based on the DNA microarray. Mol Med Rep. 2016, 13(3): 2182-6

35 D. K. Sarkar. Genesis of prolactinomas: studies using estrogen-treated animals. Front Horm Res. 2006, 3532-49

36 J. Torresani; L. J. DeGroot. Triiodothyronine binding to liver nuclear solubilized proteins in vitro. Endocrinology. 1975, 96(5): 1201-9

37 W. Pirker; M. Riedl; A. Luger; T. Czech; K. Rossler; S. Asenbaum; P. Angelberger; J. Kornhuber; L. Deecke; I. Podreka; T. Brucke. Dopamine D2 receptor imaging in pituitary adenomas using iodine-123-epidepride and SPECT. J Nucl Med. 1996, 37(12): 1931-7

38 W. Jia; A. J. Sanders; G. Jia; X. Liu; R. Lu; W. G. Jiang. Expression of the mTOR pathway regulators in human pituitary adenomas indicates the clinical course. Anticancer Res. 2013, 33(8): 3123-31

39 C. Biberger; E. von Angerer. 2-Phenylindoles with sulfur containing side chains. Estrogen receptor affinity, antiestrogenic potency, and antitumor activity. J Steroid Biochem Mol Biol. 1996, 58(1): 31-43

40 C. J. Newton; T. Trapp; U. Pagotto; U. Renner; R. Buric; G. K. Stalla. The oestrogen receptor modulates growth of pituitary tumour cells in the absence of exogenous oestrogen. J Mol Endocrinol. 1994, 12(3): 303-12

41 I. Shimon; D. R. Hinton; M. H. Weiss; S. Melmed. Prolactinomas express human heparin-binding secretory transforming gene (hst) protein product: marker of tumour invasiveness. Clin Endocrinol (Oxf). 1998, 48(1): 23-9
